# Supplementary material for: Conformational dynamics of a nicotinic receptor neurotransmitter site
Source: eLife. 2024 Dec 18;13:RP92418. doi: 10.7554/eLife.92418 (PMC11655062; doi:10.7554/eLife.92418)
Supplement: Figure 2—source data 1. — Average root-mean-square deviation (RMSD) values for protein, ligand, or the entire complex (protein + ligand) across individual MD simulation runs. SD, standard deviation; SEM, standard error of the mean. [file elife-92418-fig2-data1.docx]

|  | Time (ns) | System | RMSD (Å) | | | SD | SEM |
| --- | --- | --- | --- | --- | --- | --- | --- |
|  |  |  | Run1 | Run2 | Run3 |  |  |
| CCh | 200 | Complex | 1.84 | 2.20 | 2.19 | 0.21 | 0.12 |
|  |  | Ligand | 4.26 | 4.75 | 4.92 | 0.34 | 0.2 |
|  |  | Protein | 1.27 | 1.64 | 2.06 | 0.39 | 0.23 |
| ACh | 200 | Complex | 2.40 | 2.95 | 3.01 | 0.27 | 0.16 |
|  |  | Ligand | 5.81 | 6.83 | 5.57 | 0.53 | 0.31 |
|  |  | Protein | 1.19 | 2.44 | 2.53 | 0.27 | 0.15 |
| Ebt | 200 | Complex | 2.29 | 2.04 | 2.00 | 0.16 | 0.09 |
|  |  | Ligand | 4.37 | 3.85 | 3.08 | 0.65 | 0.37 |
|  |  | Protein | 1.14 | 1.58 | 1.55 | 0.25 | 0.14 |
| Ebx | 200 | Complex | 2.91 | 2.81 | 2.70 | 0.09 | 0.05 |
|  |  | Ligand | 5.46 | 5.59 | 5.30 | 0.09 | 0.07 |
|  |  | Protein | 2.90 | 2.22 | 2.19 | 0.33 | 0.19 |
