# Supplementary material for: Conformational dynamics of a nicotinic receptor neurotransmitter site
Source: eLife. 2024 Dec 18;13:RP92418. doi: 10.7554/eLife.92418 (PMC11655062; doi:10.7554/eLife.92418)
Supplement: Figure 3—source data 1. — The table presents the percentage of the total variance in the molecular motion that is accounted for by the first ten principal components in five different simulation conditions: Apo; CCh, carbamylcholine; ACh, acetylcholine; Ebt, epibatidine, and Ebx, epiboxidine. Each row corresponds to a principal component (PC1 through PC10), while the columns list the cumulative percentage of motion variance that the PCs account for in each simulation condition. [file elife-92418-fig3-data1.docx]

| **PCs** | **Cumulative contribution %** | | | | |
| --- | --- | --- | --- | --- | --- |
|  | **Apo** | **CCh** | **ACh** | **Ebt** | **Ebx** |
| PC1 | 20.72 | 31.98 | 49.55 | 23.25 | 30.14 |
| PC2 | 27.19 | 35.47 | 57.23 | 31.86 | 37.81 |
| PC3 | 38.49 | 45.24 | 62.15 | 40.29 | 44.01 |
| PC4 | 44.36 | 52.69 | 65.9 | 45.99 | 49.09 |
| PC5 | 48.58 | 56.38 | 69.18 | 49.93 | 53.52 |
| PC6 | 52.33 | 59.42 | 71.4 | 53.3 | 57.51 |
| PC7 | 55.67 | 61.58 | 73.06 | 56.39 | 60.17 |
| PC8 | 58.44 | 63.57 | 74.66 | 59.09 | 62.53 |
| PC9 | 60.78 | 65.39 | 76.09 | 61.45 | 64.61 |
| PC10 | 62.76 | 66.99 | 77.28 | 63.48 | 66.46 |
