# Supplementary material for: Conformational dynamics of a nicotinic receptor neurotransmitter site
Source: eLife. 2024 Dec 18;13:RP92418. doi: 10.7554/eLife.92418 (PMC11655062; doi:10.7554/eLife.92418)
Supplement: Figure 4—source data 1. — Agonists, see Figure 2A. In silico (calculated) values were from frames from each principal component analysis (PCA) population (m1, m2, and m3; Figure 3) after cluster analysis of ligand orientation. In vitro (experimental) values were from electrophysiology measurements. Window, frame times for each population; n frames, number of simulation frames from three prominent clusters chosen for MM-PBSA analysis;ΔH, change in binding enthalpy; ΔS change in binding entropy (T, absolute temperature);ΔH, change in Gibbs free energy (ΔG=ΔH-TΔS); η, efficiency (1-ΔGLA/ΔGHA). SD, standard deviation; SEM, standard error of the mean. Data are shown as bar graphs in Figure 4. [file elife-92418-fig4-data1.docx]

|  |  | **window (ns)** | **n frames** | **ΔH**  **(SD, SEM)** | **TΔS**  **(SD, SEM)** | **ΔG calculated** | **ΔG experimental** | **η calculated** | **η**  **experimental** |
| --- | --- | --- | --- | --- | --- | --- | --- | --- | --- |
| **CCh** | **m1** | 15-24 | 226 | -27.61  (7.66, 0.49) | -22.88  (5.82, 2.37) | -4.73 | -4.44 | 0.52 | 0.52 |
|  | **m2** | 100-120 | 273 | -24.41  (5.68, 0.79) | -17.54  (3.49, 1.42) | -6.87 |  |  |  |
|  | **m3** | 186-200 | 207 | -26.18  (5.75, 0.47) | -16.25  (7.76, 2.24) | -9.93 | -9.20 |  |  |
| **ACh** | **m1** | 5-14 | 100 | -20.83  (8.11, 1.13) | -14.27  (6.51, 3.25) | -6.56 | -5.11 | 0.47 | 0.50 |
|  | **m2** | 20-50 | 359 | -27.92  (3.01, 0.29) | -16.72  (2.53, 1.03) | -11.2 |  |  |  |
|  | **m3** | 160-200 | 266 | -27.00  (2.88, 0.87) | -14.57  (6.28, 3.63) | -12.43 | -10.31 |  |  |
| **Ebt** | **m1** | 21-41 | 591 | -27.64  (3.83, 0.27) | -13.42  (4.70, 1.42) | -14.22 | -6.20 | 0.42 | 0.42 |
|  | **m2** | 50-60 | 325 | -24.21  (4.25, 0.42) | -13.50  (6.81, 2.05) | -10.71 |  |  |  |
|  | **m3** | 175-200 | 996 | -41.77  (3.99, 0.28) | -17.23  (6.86, 2.07) | -24.54 | -10.60 |  |  |
| **Ebx** | **m1** | 20-56 | 1284 | -20.51  (2.98, 0.46) | -12.85  (5.49, 1.66) | -7.66 | -5.40 | 0.48 | 0.46 |
|  | **m2** | 60-100 | 504 | -21.55  (3.56, 0.28) | -14.39  (10.46, 3.15) | -7.16 |  |  |  |
|  | **m3** | 174-200 | 562 | -33.95  (2.92, 0.88) | -19.33  (4.39, 1.32) | -14.62 | -9.96 |  |  |
