# Supplementary material for: Conformational dynamics of a nicotinic receptor neurotransmitter site
Source: eLife. 2024 Dec 18;13:RP92418. doi: 10.7554/eLife.92418 (PMC11655062; doi:10.7554/eLife.92418)
Supplement: Figure 4—source data 2. — EEL, electrostatic; VdW, van der Waals; EPB, electrostatic Poisson-Boltzmann; ENPOLAR, nonpolar solvation energy; ΔG gas, gas-phase free energy; ΔG solv, solvation free energy; SD, standard deviation; SEM, standard error of the mean. [file elife-92418-fig4-data2.docx]

|  |  | EEL | Vdw | EPB | ENPOLAR | ΔG gas | ΔG solv |
| --- | --- | --- | --- | --- | --- | --- | --- |
| CCh | m1 | -333.79 (11.75, 0.76) | -21.83  (2.63, 0.17) | 330.28 (12.61, 0.81) | -2.17  (0.08, 0.01) | -355.72 (11.88, 0.76) | 328.10  (12.57, 0.81) |
|  | m2 | -320.28 (11.95, 1.67) | -25.53  (3.28, 0.46) | 323.63 (11.34, 1.58) | -2.23  (0.12, 0.01) | -345.81 (13.66, 1.91) | 321.40  (11.36, 1.59) |
|  | m3 | -328.51  (8.58, 0.69) | -30.64  (2.12, 0.17) | 335.07  (7.34, 0.59) | -2.08  (0.08, 0.01) | -359.15  (9.23, 0.74) | 332.98  (7.35, 0.59) |
| ACh | m1 | -304.06 (11.36, 3.88) | -22.44  (5.56, 0.78) | 305.64 (12.44, 3.75) | -2.21  (0.06, 0.02) | -324.26 (17.16, 5.17) | 303.43  (12.43, 3.74) |
|  | m2 | -311.50  (7.67, 0.76) | -27.94  (1.88, 0.19) | 313.74  (6.63, 0.66) | -2.23  (0.07, 0.01) | -339.43  (7.89, 0.78) | 311.51  (6.65, 0.66) |
|  | m3 | -292.97  (5.14, 1.55) | -29.15  (1.96, 0.59) | 297.33  (4.66, 1.40) | -2.23  (0.05, 0.01) | -322.10  (5.71, 1.72) | 295.10  (4.67, 1.4101) |
| Ebt | m1 | -354.29 (13.62, 0.96) | -28.18  (2.13, 0.15) | 357.44 (13.74, 0.96) | -2.62  (0.07, 0.01) | -382.46 (13.82, 0.97) | 354.82  (13.73, 0.9691) |
|  | m2 | -349.73 (10.40, 1.03) | -29.63  (2.54, 0.25) | 357.78  (9.80, 0.97) | -2.62  (0.08, 0.01) | -379.36 (10.46, 1.04) | 355.15  (9.80, 0.97) |
|  | m3 | -359.83  (7.83, 0.55) | -37.95  (1.95, 0.14) | 358.39  (7.13, 0.50) | -2.38  (0.04, 0.00) | -397.77  (7.74, 0.54) | 356.01  (7.13, 0.50) |
| Ebx | m1 | -319.00  (6.39, 0.99) | -27.13  (2.14, 0.33) | 328.16  (7.44, 1.16) | -2.55  (0.07, 0.01) | -346.13  (6.83, 1.06) | 325.61  (7.46, 1.16) |
|  | m2 | -321.95 (15.34, 1.21) | -23.34  (2.34, 0.18) | 326.33 (14.61, 1.15) | -2.61  (0.10, 0.01) | -345.26 (16.18, 1.27) | 323.71  (14.62, 1.15) |
|  | m3 | -321.11  (8.45, 2.55) | -30.67  (1.69, 0.51) | 320.25  (8.43, 2.54) | -2.43  (0.06, 0.01) | -351.77  (9.18, 2.76) | 317.81  (8.43, 2.54) |
