# Supplementary material for: Conformational dynamics of a nicotinic receptor neurotransmitter site
Source: eLife. 2024 Dec 18;13:RP92418. doi: 10.7554/eLife.92418 (PMC11655062; doi:10.7554/eLife.92418)
Supplement: Figure 6—source data 1. — Distance measurements (Å) between functional groups. αK145 is to the companion polar group; N+ is to the center of the indole ring; loop C is Cα of αC192 compared apo; m1, m2, and m3 structures taken from plateau regions of the trajectories. [file elife-92418-fig6-data1.docx]

|  |  | αK145-αD200 | αK145-αY190 | αD200-αY190 | N^+^-αW149 | loop C (αC192) |
| --- | --- | --- | --- | --- | --- | --- |
| CCh | m1 | 6.1 | 9.3 | 7.8 | 5.4 | 2.7 |
|  | m2 | 6.3 | 9.5 | 7.9 | 4.5 | 3.8 |
|  | m3 | 2.6 | 6.1 | 4.7 | 4.2 | 12.1 |
| ACh | m1 | 5.1 | 7.8 | 6.3 | 4.6 | 3.7 |
|  | m2 | 3.1 | 7.0 | 7.6 | 4.8 | 7.4 |
|  | m3 | 2.8 | 5.3 | 4.4 | 4.4 | 9.6 |
| Epi | m1 | 3.4 | 14.7 | 14.0 | 5.1 | 6.8 |
|  | m2 | 3.7 | 5.8 | 6.2 | 5.0 | 7.5 |
|  | m3 | 4.4 | 6.7 | 6.1 | 3.3 | 7.9 |
| Ebx | m1 | 4.4 | 8.5 | 7.8 | 6.3 | 5.5 |
|  | m2 | 3.8 | 9.7 | 8.3 | 5.4 | 4.7 |
|  | m3 | 5.4 | 8.0 | 5.2 | 3.8 | 9.7 |
